# Assessment of quality of primary care with facility surveys: a descriptive analysis in ten low-income and middle-income countries

**DOI:** 10.1016/S2214-109X(18)30440-6

**Published:** 2018-10-12

**Authors:** Erlyn K Macarayan, Anna D Gage, Svetlana V Doubova, Frederico Guanais, Ephrem T Lemango, Youssoupha Ndiaye, Peter Waiswa, Margaret E Kruk

**Affiliations:** a Department of Global Health and Population, Harvard T H Chan School of Public Health, Boston, MA, USA; b Ariadne Labs, Brigham and Women's Hospital and Harvard T H Chan School of Public Health, Boston, MA, USA; c Epidemiology and Health Services Research Unit, Mexican Institute of Social Security, Mexico City, Mexico; d Division of Social Protection and Health, Inter-American Development Bank, Lima, Peru; e Maternal and Child Health Directorate, Federal Ministry of Health, Addis Ababa, Ethiopia; f Planning, Research, and Statistics, Ministry of Health and Social Action, Dakar, Senegal; g Department of Public Health Sciences—Global Health, Karolinska Institute, Stockholm, Sweden; h Maternal and Newborn Centre of Excellence, Makerere University School of Public Health, Kampala, Uganda; i INDEPTH Network Maternal and Newborn Working Group, Iganga-Mayuge Health and Demographic Surveillance Site, Iganga, Uganda

## Abstract

**Background:**

Primary care has the potential to address a large proportion of people's health needs, promote equity, and contain costs, but only if it provides high-quality health services that people want to use. 40 years after the Declaration of Alma-Ata, little is known about the quality of primary care in low-income and middle-income countries. We assessed whether existing facility surveys capture relevant aspects of primary care performance and summarised the quality of primary care in ten low-income and middle-income countries.

**Methods:**

We used Service Provision Assessment surveys, the most comprehensive nationally representative surveys of health systems, to select indicators corresponding to three of the process quality domains (competent systems, evidence-based care, and user experience) identified by the Lancet Global Health Commission on High Quality Health Systems in the Sustainable Development Goals Era. We calculated composite and domain quality scores for first-level primary care facilities across and within ten countries with available facility assessment data (Ethiopia, Haiti, Kenya, Malawi, Namibia, Nepal, Rwanda, Senegal, Tanzania, and Uganda).

**Findings:**

Data were available for 7049 facilities and 63 869 care visits. There were gaps in measurement of important outcomes such as user experience, health outcomes, and confidence, and processes such as timely action, choice of provider, affordability, ease of use, dignity, privacy, non-discrimination, autonomy, and confidentiality. No information about care competence was available outside maternal and child health. Overall, scores for primary care quality were low (mean 0·41 on a scale of 0 to 1). At a domain level, scores were lowest for user experience, followed by evidence-based care, and then competent systems. At the subdomain level, scores for patient focus, prevention and detection, technical quality of sick-child care, and population-health management were lower than those for other subdomains.

**Interpretation:**

Facility surveys do not capture key elements of primary care quality. The available measures suggest major gaps in primary care quality. If not addressed, these gaps will limit the contribution of primary care to reaching the ambitious Sustainable Development Goals.

**Funding:**

Bill & Melinda Gates Foundation.

## Introduction

Primary care has great potential to improve population health outcomes through early intervention in the disease process and coordinated provision of care. Strong primary care systems are associated with reduced morbidity, increased patient longevity, and increased equity in health outcomes in several countries.^1^, ^2^ However, the standard model whereby primary care is the first point of contact for most health needs is being challenged by rapid urbanisation, which results in a greater choice of providers, the growth of unregulated private providers, shifts in epidemiology that change the profile of the typical patient needing primary care, and people's increasing expectations for highly effective care.^3^, ^4^, ^5^ As the world marks 40 years since the Alma-Ata Declaration, little is known about the functioning of primary care in low-income and middle-income countries (LMICs).

Most research about primary care performance has been done in high-income settings, and has been based on metrics that were not available in LMICs. Many of the studies^6^, ^7^, ^8^, ^9^ of measures of primary care done in LMICs have focused on specific health conditions and services, such as quality of family planning and sick-child care. Other studies focused on input-based^9^ (eg, number of beds or health workers) and aggregated outcome-based^10^ (eg, health-care access and quality index) measures. Input-based measures provide little insight into the practice of providers, whereas aggregated outcome-based measures can be confounded by the degree and severity of patients' clinical conditions.^11^ If quality of care is measured on the basis of outcomes only, providers might avoid handling patients with worse conditions because treatment of such high-risk patients would be likely to result in lower quality scores.^11^ Furthermore, these aggregated outcomes-based measures do not identify specific areas for improvement. Thus, exclusive focus on outcomes results in inadequate identification of disparities between access to care and quality of care received from providers. Process measures, which are based on the encounter between the patient and a health-care professional (eg, diagnoses, treatment, referral, prescribing),^12^ are thus a valuable component of assessments of primary care. Although assessment of process-based measures in primary care in LMICs is crucial to track progress, too often the focus has been on access rather than on quality of care measures.

**Research in context**

**Evidence before this study**

Evidence for this study was gathered via a scoping review of published literature and comprehensive collection of available survey datasets examining the quality-of-care measures relevant for primary care according to the *Lancet Global Health* Commission framework (a detailed description of the search is included in the appendix). Previous cross-national studies of the quality of primary care in low-income and middle-income countries (LMICs) focused mainly on measures that were input-based (eg, number of beds or health workers) or outcome-based (eg, mortality attributable to primary care, avertable hospitalisations). Data for patients' experiences were gathered in population surveys in several Latin American countries, but these surveys did not include objective assessments of care competence. Furthermore, previous studies focused on technical quality rather than capturing a broad view of primary care systems.

**Added value of this study**

We applied the *Lancet Global Health* Commission on High Quality Health Systems in the Sustainable Development Goals Era framework to primary care settings to identify how well facility surveys capture important data for the quality of primary care and to establish what available data show about quality of primary care in LMICs. We identified few indicators of user experience, health outcomes, or confidence, and no process measures on timely action, choice of provider, affordability, ease of use, dignity, privacy, non-discrimination, autonomy, or confidentiality. For evidence-based care, indicators were available only for three health conditions—sick-child care, family planning, and antenatal care—and not for other conditions that could benefit from primary care (eg, hypertension, diabetes, depression, respiratory disease). Overall, scores for the quality of primary care in LMICs were low.

**Implications of all the available evidence**

Our findings show gaps in the measurement of primary care quality and suggest the need for new measures and instruments. They also provide empirical evidence to guide future monitoring and assessment of the performance of primary care in LMICs. Variations in primary care quality at subnational and national levels can inform strategies for equitable delivery of primary care services.

Facility surveys are data collection instruments for surveying facilities. Facility surveys have been designed by WHO and the World Bank, among others. Prominent examples are the Service Provision Assessment, Service Availability and Readiness Assessment, and Service Delivery Indicators. They can include direct observations of care, interviews, and clinical and patient vignettes. Facility surveys are usually used to improve understanding of the supply side of health care. They can also be used to measure the quality of primary care processes, but little is known about this process. Furthermore, in broader assessments of health-systems performance, hospitals and large health facilities tend to be over-represented compared with low-level facilities that often provide the first contact care.

We aimed to address these gaps by using a framework proposed by the *Lancet Global Health* Commission on High Quality Health Systems in the Sustainable Development Goals Era^13^ (HQSS) to examine the measurement and performance of primary care platforms in LMICs. The Commission's framework was developed to provide an updated approach to measurement of health-system performance that emphasises value to people (appendix), and was informed by other primary care and health-system frameworks.^14^, ^15^, ^16^, ^17^ The framework consists of three main domains: foundations, processes of care, and quality effects. In this study, we focused on the processes-of-care domain (which has been called the “black box” of primary care^17^). We used the most comprehensive published facility surveys in LMICs to identify gaps in primary care measurement, then we developed a quality score in countries with available data, and used these measures to examine variations in quality as it pertained to processes of care at the national and subnational level. Our hope was that these findings would serve as baseline evidence for the identification of necessary additional measures and simplify existing measures of quality of care, which could eventually be linked with patient outcomes.

## Methods

### Data sources and variables

We used the most recent Service Provision Assessment (SPA)^18^ facility survey data from 2007 to 2016 in our analyses. The SPA is a nationally representative health-facility assessment that includes a facility assessment, a questionnaire for health-care providers, observations of visits, and exit interviews with observed patients.^18^ We selected SPA surveys for this analysis because they are the most comprehensive, standardised, cross-nationally available datasets of health-system measurements.^18^ Other global facility surveys include WHO's Service Availability and Readiness Assessment surveys,^19^ which focus mainly on infrastructure and equipment, and the World Bank's Service Delivery Indicators surveys,^20^ which measure the knowledge of health providers and health facility resources. However, neither of these surveys captures the process of care that people receive.

We included only the ten countries with available data: Ethiopia, Uganda, Senegal, Nepal, Kenya, Tanzania, Rwanda, Malawi, Haiti, and Namibia. SPAs, are done differently across countries. Ethiopia, Uganda, Senegal, Nepal, Kenya, and Tanzania had nationally representative survey data, whereas Rwanda, Malawi, Haiti, and Namibia had national censuses.^18^ Although a SPA survey was done in Bangladesh in 2014, we did not include it because it did not contain any observations of visits, which were required for many of the measures we defined. We wanted to use survey data from 2007 to 2016 only to approximate the contemporary situation, although we recognise that many changes might have occurred since some of the older surveys.

In the survey countries, facility sampling weights were used to correct for oversampling of hospitals and to create health-system representative estimates, and providers and clients were randomly sampled within facilities on the day of the survey.^18^ Typically, SPA surveys collect data from 400–700 facilities selected at random from a comprehensive list of health facilities in a country.^18^ Hospitals can be oversampled because there tend to be only small numbers of hospitals in a country.^18^ Subsequently, the data were weighted during analysis to ensure that data were proportionally representative when presented (appendix).^18^

We limited our analysis to primary care facilities, which include the first level of care from health centres, clinics and polyclinics, health posts, dispensaries, and other low-level facilities. Although primary care services can be provided at hospitals, this assessment was designed to provide a cross-nationally comparable view of care quality at the first level of care. Thus, we removed hospital primary care from the analysis, but still weighted the facilities to ensure that the analyses were nationally representative of primary care facilities in the study countries.

To provide country context corresponding to the SPA survey year, we obtained data for gross domestic product per person, Gini index, health expenditure per person, and number of health workers (community health workers, physicians, and nurses) per 100 000 people, land area, and proportion of urban areas for each country from the World Development Indicators.^21^

### Identification of gaps in quality measurement

We adapted the Commission framework to focus on primary-health-care systems. The four Cs of primary care—continuous, coordinated, first contact, and comprehensive care—were mapped to the three main domains of the processes of care: competent systems, evidence-based care, and positive user experience (table 1).^13^ Competent systems were composed of the subdomains safety, prevention and detection, continuity and integration, population-health management, and timely action. Evidence-based care included systematic assessment, correct diagnosis, appropriate treatment, and counselling, and were assessed for key primary care services (antenatal care, family planning, sick-child care, non-communicable diseases, mental health, HIV, tuberculosis, and other primary-care-sensitive conditions [ie, conditions for which good primary care could prevent the need for hospital admission, or for which early intervention could prevent complications or more severe disease—eg, angina, asthma, chronic obstructive pulmonary disease, congestive heart failure, diabetes mellitus, hypertension^10^]). Positive user experience was composed of patient focus—which included short wait times and patient voice and values—and clear communication.

**Table 1:** Mapping of primary care indicators to Commission framework

	**Descriptions specific to primary care**
**Competent systems**	
Safety	Primary care systems seek to prevent harm to patients by ensuring facility cleanliness and that safety precautions and other safety interventions (eg, sterilisation, sharp and waste disposal, infection-control items) are in place. An unsafe primary care system predisposes patients to adverse events and injuries due to medical devices and injuries due to surgical and anaesthesia errors, including wrong-site surgery, health-care-associated infections, improper transfusion and injection practices, falls, burns, and pressure ulcers.
Prevention and detection	The prevention and early detection of diseases, including through screening when indicated or referrals when needed, are important functions of high-quality health systems, especially primary care systems.
Continuity and integration	Continuity of care is shown by the health system's ability to retain people in care, and, for the patient, by their ability to see a clinician familiar with their medical history. Integration is the extent to which health services are delivered in a complementary and coherent manner. Scheduling of follow-up visits and tracking of care with vaccination cards and client records are some examples of ensuring continuous and integrated primary care systems.
Population-health management	Population-health management, such as outreach services and community meetings, is core to primary care systems, in which data for the patient population should be collected, analysed, and acted upon to optimise how to best manage specific diseases within that population.
Timely action	Timely actions in primary care systems optimise patient outcomes and reduce the need for additional admissions because of complications arising from service provision. Timeliness is also crucial for conditions that can be cured if treated early, including many cancers and conditions such as tuberculosis or diabetes, for which early treatment prevents transmission or disease progression. For people with life-threatening emergencies, such as labour complications, trauma, and strokes, treatment delays substantially increase mortality risk.
**Evidence-based care**	
Technical quality indices for key primary care services*	Evidence-based care is exhibited when there is systematic assessment, correct diagnosis, appropriate treatment, and counselling. A systematic patient assessment involves gathering clinically relevant information by asking appropriate history questions and doing the recommended examinations and tests. Incorrect diagnoses have deleterious effects on health and contribute to treatment delays and antimicrobial resistance. Treatments should be appropriate: underuse of effective care and overuse of unnecessary care lead to primary care of poor quality. Proper counselling and client education are essential elements of evidence-based care. For example, during antenatal care, many skilled providers do not advise women about the signs of pregnancy complications or how to prevent HIV infection, and, when prescribing contraceptives, many do not discuss their potential side-effects.
**Positive user experience**	
Patient focus	Providers have shown care that is respectful of, and responsive to, individual patient preferences, needs, and values.
Clear communication	Clear communication is shown when providers have adequately explained and discussed care plans and treatment processes such as follow-up visits and use of family-planning methods and their side-effects or other danger signs.

* See appendix for specific indicators under evidence-based care for each type of service.

To identify the indicators, two authors (EKM and ADG) individually assessed the list of indicators from the SPA datasets and, on the basis of the HQSS framework, identified and classified indicators relevant to measurement of quality of care. Individual assessments were cross-checked through group discussion to ensure consistency of classification. In the cases of discrepancy, a third researcher (MEK) participated to corroborate the domain and subdomain of each indicator.

### Calculation of quality scores

Three types of score were calculated for each facility: subdomain scores (mean of component indicators relevant for each subdomain), domain scores (mean of the nine subdomain scores), and overall quality of primary care scores (mean of the three domain scores). All index component indicators were either binary (ie, 0 or 1) or indexes ranging from 0 (lowest) to 1 (highest). If the indicators were at the patient level, such as for evidence-based care and user experience indicators, the average was calculated to get the mean scores for each facility. For evidence-based care, the average technical quality indices were calculated by averaging the client-level scores for systematic assessment, correct diagnosis, appropriate treatment, and counselling for every visit in the primary care facility. To calculate for the technical quality indices, process indicators specific to each type of primary care service were selected from SPA.^22^ These binary indicators, ranging from 0 to 1, were then averaged to create a score for technical quality for each service. These indices defined technical quality of care in each service by identifying key domains of care and the essential clinical actions within each domain from international guidelines.^22^ These domains included history, examination, and counselling. Antenatal care and sick-child care included items on testing and management.^22^ The list of indicators for each technical quality index is in the appendix.

### Statistical analysis

All patient-level analysis included SPA client survey weights.^18^ The overall quality scores, and scores for the domain and subdomains were calculated on the basis of facility survey weights.^18^ We weighted each country equally when averaging scores across countries because our goal was to generalise across countries instead of across populations. We then compared scores at national and subnational levels. Scores were mapped with the Database of Global Administrative Areas and QGIS (version 2.18). Correlations between quality scores and several national-level predictors were calculated. All analyses were done in Stata (version 15.0), which was also used to plot the figures (except for the coxcombs, which were made in Vizzlo).

### Role of the funding source

The study funders had no role in study design; data collection, analysis, or interpretation; or writing of the report. The corresponding author had full access to all the data in the study and had final responsibility for the decision to submit for publication.

## Results

In our gap analysis, we identified few indicators for the HQSS subdomains user experience, health outcomes, or confidence, and no process measures of timely action, choice of provider, affordability, ease of use, dignity, privacy, non-discrimination, autonomy, and confidentiality. For evidence-based care, indicators were available only for three health conditions: sick-child care, family planning, and antenatal care. We did not identify indicators for other conditions that could benefit from primary care, such as hypertension, diabetes, depression, or respiratory disease.

Of the 8274 facilities surveyed in SPAs, 7049 (85·2%) were primary care facilities (unweighted; table 2). Our sample included 15 269 antenatal-care visits, 25 447 family-planning visits, and 23 153 sick-child-care visits. Of the primary care facilities surveyed in the five countries with available data for facility location, 47% were in an urban area (table 2). The mean service readiness index score, which measured facilities' capacity to deliver health services on a scale of 0 to 1, on which 1 indicates greater readiness, was 0·53 (table 2).

**Table 2:** Characteristics of primary care facility and country contexts, by survey year

			**Overall**	**Ethiopia (2014)**	**Haiti (2013)**	**Kenya (2010)**	**Malawi (2013)**	**Namibia (2009)**	**Nepal (2015)**	**Rwanda (2007)**	**Senegal (2015–16)**	**Tanzania (2015)**	**Uganda (2007)**
Primary care facilities (n)			7049	1104	786	443	941	366	722	496	882	937	372
Visits observed (n)													
	Antenatal care		15 269	1853	1620	1409	2068	859	1509	722	849	4007	373
	Family planning		25 447	3100	2922	2416	3567	1838	2281	1395	1718	5753	457
	Sick-child care		23 153	1908	2442	2016	3329	1544	2186	1709	2289	4961	769
Study facility characteristics*													
	Managing authority (%)												
		Public	4532 (64%)	625 (57%)	294 (37%)	195 (44%)	458 (49%)	290 (79%)	671 (93%)	284 (57%)	752 (85%)	674 (72%)	289 (78%)
		Private non-profit	888 (13%)	21 (2%)	312 (40%)	87 (20%)	183 (19%)	37 (10%)	51 (7%)	0 (0%)	83 (9%)	114 (12%)	0 (0%)
		Private for-profit	1629 (23%)	458 (41%)	180 (23%)	161 (36%)	300 (32%)	39 (11%)	0 (0%)	212 (43%)	47 (5%)	149 (16%)	83 (22%)
	Location (%)												
		Urban	3138 (45%)	680 (62%)	528 (67%)	..	667 (71%)	..	..	..	594 (67%)	669 (71%)	..
		Rural	1507 (21%)	424 (38%)	258 (33%)	..	274 (29%)	..	..	..	283 (32%)	268 (29%)	..
	Mean service readiness index†		0·53	0·35	0·52	0·55	0·55	0·68	0·44	0·59	0·57	0·48	0·41
Country context													
	Regions (subnational levels; n)		108	11	10	8	3	13	5	5	14	30	9
	Gross domestic product per person (US$)‡		2464	1501	1686	2426	1099	7854	2469	1091	2571	2652	1291
	Gini index		43	33	41	49	46	61	33	51	40	38	43
	Health expenditure per person (US$)		165	73	136	99	87	628	137	91	107	137	155
	Health workers per 100 000 population (n)												
		Physicians	12	2	26	18	2	37	21	5	..	3	12
		Nurses and midwives	62	24	70	74	28	278	47	67	42	43	134
		Community health workers	51	36	..	..	72	..	68	136	..	..	19
	Land area (per 100 m^2^)		40	100	3	57	9	82	14	2	19	89	20
	Urban population (% of total)		28	19	56	24	16	41	19	21	44	32	14

Data are our analysis of Service Provision Assessments. Country context data were from the World Development Indicators and the World Bank report for Haiti,^14^ for the years corresponding to the Service Provision Assessment survey years. Sample includes unweighted numbers of observations. Primary care facilities focus on first-level care, and include health centres, clinics, polyclinics, health posts, dispensaries, and other low-level facilities.

* Facility characteristics were calculated with facility survey weights.

† Service readiness index refers to the overall capacity of health facilities to provide general health services; readiness is defined as the availability of components required to provide services such as basic amenities, basic equipment, standard precautions, laboratory tests, and medicines and commodities (values closer to 1 indicate greater readiness); data are from WHO's Service Availability and Readiness Assessment.

‡ In purchasing power parity or expenditure-weighted averages of relative prices of a vast number of goods and services on which people spend their incomes.

Of about 900 SPA indicators, we identified 126 that could be used to measure quality of primary care. From these 126 indicators, we identified 37 that corresponded to the three process of care domains and nine subdomains in the Commission (panel; appendix). For the competent systems domain, indicators included facility cleanliness, sterilisation and waste-disposal activities, screening and referrals, counselling services, outreach, and staff community meetings. For the evidence-based-care domain, indicators included systematic assessments (eg, blood pressure checks, measurement of weight and height), diagnosis, treatment (eg, tests, referrals), and counselling services for antenatal care, family planning, and sick-child care. For the user experience domain, indicators included systems for discussing patient preferences and reviewing patient opinions, waiting times, patient knowledge of their treatment, counselling, and provider explanations. To contextualise the primary care measurements across each country, scores for each domain, subdomain, and component indicator across the study countries are in the appendix.

Panel **Quality of primary care component indicators selected from the Service Provision Assessment survey**

Competent systems*

*Safety*

• Kept the facility clean
• Sterilised used equipment
• Disposed sharps adequately
• Observed guideline for standard precautions
• Proportion of sharps and medical or contaminated waste adequately disposed of, and availability of waste-disposal guidelines*
• Proportion of rooms with all infection control items (water, soap, hand disinfectant, gloves, surface disinfectant, and sharp-disposal system)*

*Prevention and detection*

• Provided tuberculosis screening and counselling for patients with HIV
• Provided HIV tests for patients with tuberculosis
• During antenatal care, facility did or referred the patient for
  • Urine test
  • Syphilis test
  • HIV counselling and testing

*Continuity and integration*

• Proportion of core primary care services offered (care for sick children, immunisation, growth monitoring, antenatal care, family planning, HIV, tuberculosis, and malaria)*
• Proportion of services offered for additional common non-communicable disease (eg, diabetes, cardiovascular diseases, chronic renal disease)*
• Checked vaccination card for sick child
• Counselled patient about postnatal family planning
• Discussed follow-up visits for family planning
• Proportion of antenatal-care, family-planning, sick-child, and sexually-transmitted-disease services for client records are kept*
• Proportion of services with test-return agreements*

*Population-health management*

• Offered all core outreach services (care for sick children, growth monitoring, and immunisation services) at least once a month
• Held staff community meetings

**Evidence-based care**

*Technical quality*

Indices for observing systematic assessment, correct diagnosis, appropriate treatment, and counselling during provision of care (appendix):

• Component measure from 18 antenatal-care indicators*
• Component measure from 33 family-planning indicators*
• Component measure from 20 sick-child indicators*

**Positive user experience**

*Patient focus*

• Uses systems for discussing patient preferences
• Uses systems for reviewing or reporting patient opinions
• Proportion of wait times less than 1 h*

*Clear communication*

During antenatal visit, patient knows about:

• Delivery preparation
• Childbirth complications
• Side-effects of iron
• At least one danger sign

During family planning visit, provider explained:

• Use of family-planning method
• Family-planning side-effects,
• Follow-up visits
• Danger signs or needed actions

During sick child visit:

• Provider communicated diagnosis
• Provider recommended food or liquid intake
• Parents or guardians felt confident about dosing and duration of drugs

**Figure 1:**
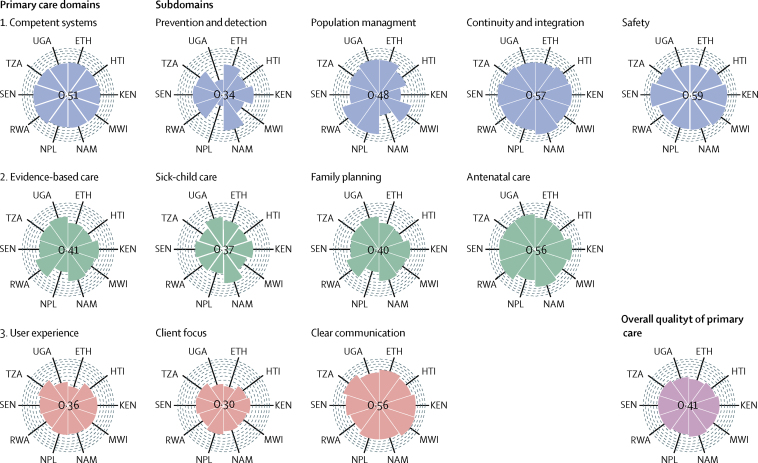
Average quality domain and subdomain scores of primary care facilities in ten low-income and middle-income countries Each arc represents an incremental score of 0·1 on a 0–1 scale. The overall quality score is the average of the scores in the domains of competent systems, evidence-based care, and user experience, which in turn are the averages of the scores in each respective subdomain (the score for evidence-based care is the average of technical quality indices for each of the subdomains). ETH =Ethiopia. HTI=Haiti. KEN=Kenya. MWI=Malawi. NAM=Namibia. NPL=Nepal. RWA=Rwanda. SEN=Senegal. TZA=Tanzania. UGA=Uganda.

We found low overall quality scores and varying quality at the domain and subdomain levels of processes of care in the ten LMICs included in our study (figure 1; appendix). The mean overall score for primary care quality was 0·41, ranging from 0·32 in Ethiopia to 0·46 in Namibia. At the domain level, user experience scored least (mean 0·36) followed by evidence-based care (0·41) and competent systems (0·51). At the subdomain level, client focus (0·30), prevention and detection (0·34), and technical quality of sick-child care (0·37) scored the lowest (figure 1). Uganda and Nepal scored least in prevention and detection, whereas Namibia had the highest scores. However, Namibia had a low score for population-health management compared with Rwanda and Nepal. Most countries scored high on continuity and integration and safety subdomains. Sick-child care scored especially lowly in Nepal and Malawi, and Nepal also had the lowest scores for family planning and antenatal care. For user experience subdomains, all countries—especially Ethiopia and Uganda—scored lower in client focus than in client communication.

**Figure 2:**
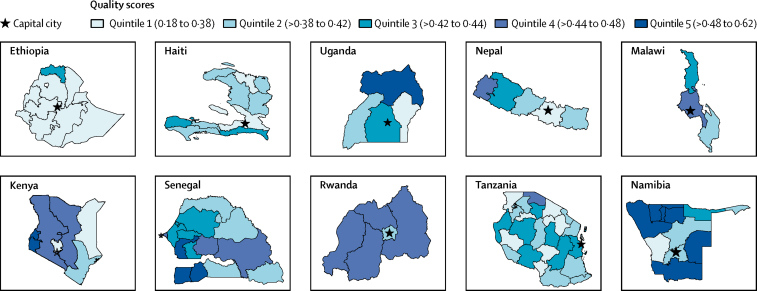
Quintiles of mean quality scores at the subnational level in study countries Base maps are from the Database of Global Administrative Areas. Quintiles are based on mean scores on overall quality of primary care for each subnational unit.

At the component indicator level, facilities scored least on systems for reporting or reviewing patient opinion (0·10), postnatal family-planning counselling (0·17), and provision of nutrition recommendations (0·21). Countries scored highest in provision of counselling during family-planning follow-up visits (0·93) and ensuring patient's knowledge of delivery preparation (0·91; appendix). At subnational levels, quintiles of regional primary care quality ranged from 0·18 in Gambela, Ethiopia, to 0·62 in Omakeke, Namibia (figure 2).

## Discussion

Although SPA is a comprehensive facility survey, in this study we showed that the quality of data and the indicators available require further development. Specifically, we identified material gaps in measurement of primary care processes in LMICs in the most comprehensive available facility surveys. There were few indicators of user experience, health outcomes, or confidence, and no process measures for timely action, choice of provider, affordability, ease of use, dignity, privacy, non-discrimination, autonomy, or confidentiality. For evidence-based care, indicators were available only for sick-child care, family planning, and antenatal care. Our findings show that assessments of primary care facilities have a much narrower focus than health-systems performance assessments, and draw attention to the need to expand to other relevant domains and subdomains to adequately assess the quality of primary care processes.

The new WHO draft declaration^23^ on primary health care highlights the insufficient and uneven implementation of primary care among and within countries, and calls for better strategies for monitoring and assessment of primary health care at national, subnational, and service-delivery levels as a complement to the Sustainable Development Goals. In this study we expanded on previous studies of the quality of primary care by using the Commission on HQSS framework, which provided a more holistic and integrative view of the quality of primary care in the studied countries. We identified poor congruence between measures from existing facility surveys and important quality measures, suggesting that current metrics are not well suited to assessment of the state and progress of primary care quality in LMICs. The corollary is that only a small proportion of the measures in the extensive SPA facility survey were relevant to our assessment, suggesting that the survey is inefficient for the purpose of gauging quality. For example, although not the focus of this study, the SPA surveys record many input measures but almost no outcome measures, even though inputs are poorly correlated to outcomes.^24^ Furthermore, linking household surveys that provide more information about patient characteristics and experiences with facility assessments would have provided a more comprehensive picture of health-care quality. The connection of both surveys would have allowed for assessment of patient characteristics and experiences (from household surveys) and health services characteristics (from facility surveys) to obtain more integrated information for assessment of quality of care.

The SPA survey focuses on reproductive, maternal, and child health services (ie, antenatal care, family planning, and sick-child care). These services were thus over-represented in the index. Measures of quality of care for non-communicable diseases, mental health, and other primary care sensitive conditions are urgently needed. Surveys should also include quality subdomains for measures such as timely action, choice of provider, affordability, ease of use, dignity, privacy, non-discrimination, autonomy, and confidentiality. Another area to examine is integrated primary care systems, about which little can be gleaned by using existing facility surveys. A key consideration in expansion of facility surveys is the potential overburdening of facility administrators and others involved in data collection. A potential next step is to discuss these measurement gaps identified in the SPA with policy makers and other key stakeholders to examine which measures of the quality of primary care to retain and which to add, with the aim of coming up with a meaningful but contained set of measures.

Scores for the overall quality of primary care were low, ranging from 0·32 in Ethiopia to 0·46 in Namibia. Scores were lowest for user experience, then evidence-based care, then competent systems. At the subdomain level, scores for patient focus, prevention and detection, technical quality of sick-child care, and population-health management were lower than those for other subdomains. This finding is echoed by those in a study^12^ done in China, Ghana, India, Mexico, Russia, and South Africa, in which one of the areas of worst performance was prevention and management of chronic conditions—eg, hypertension control, coverage of cancer screening. These findings are important for the identification of primary care domains and subdomains that need the most attention for improvement. A focus on positive user experiences and competent care that emphasises people's needs and preferences is crucial to ensure the appropriate use of health service, increased patient adherence to prescribed treatments, and better trust in health systems and health outcomes.

We also examined variations at the national and subnational level. Although Namibia had the highest overall quality score, it also had the most varied regional quality scores (followed by Uganda and Senegal). These disparities should be further explored. Subnational differences in equity require further investigation into context, governance, and funding to establish the reasons for variation.

Our study had several limitations. First, our performance indices provided only a partial view of the quality of primary care. The analysis should be treated as a starting point for identification of additional necessary measures and replacement of less useful ones, resulting in streamlining of the list of measures necessary to assess quality of care. Such approaches could help to prevent further overburdening of staff at health facilities with surveys and assessments—and health monitoring and assessment departments that do such surveys—by stopping the use of low-utility measures. Thus, findings should be updated when new measures become available. Future iterations of this study could involve decision frameworks and surveys of policy makers to examine which data should be collected by countries. Second, the primary-care quality index used in our study has not been validated or tested. Once our current index is augmented with additional elements when future survey tools are made available and adapted to national needs and priorities, our next step will be to validate the index in specific LMICs. Third, we used a simple summative measure to combine indicators because it was easier to interpret and useful for policy making. Although we could have used more complex statistical analyses, such as factor analysis, these techniques are difficult to interpret. Fourth, we limited our analysis to first-level facilities (eg, clinics, health centres) and did not include hospital-based primary care so that we could focus on the first and most accessible level of the health system. Although primary care services offered in hospitals could be better than those offered at first-level facilities, such a finding has not been consistently shown in previous studies.^4^, ^25^, ^26^ Fifth, the quality of primary care processes might even be lower than that reported if the source of the data is provider reports.^27^, ^28^ Thus, we included more measures based on observations of patient care rather than on provider reports. The former was more effective for assessment of compliance with standard practice guidelines.^29^ Finally, all technical quality indices were assessed according to well known practice guidelines, if available. For sick-child care, indices were calculated according to the Integrated Management of Childhood Illness guidelines^30^ for what should be done in every sick-child visit. For antenatal care and family planning, indices were calculated on the basis of WHO guidelines for minimum level of care. Hence, although SPAs did not allow for assessment of diagnostic accuracy or appropriateness of treatment provided, the findings were based on the minimum guidelines and standards for care delivery that can form the basic measures for assessments of quality. In view of all these limitations, our analysis should be interpreted as a starting point for robust future assessment of the quality of primary care.

Nonetheless, we have provided additional evidence about measurement of the quality of primary care with SPA surveys. SPAs are based on patient observations, which is not done in any other multicountry surveys. Although patient observations may lead to Hawthorne effects, a previous study^31^ showed that exclusion of the first observed visit for each provider did not materially change the interprovider variation of quality. Even though there could be potential bias issues, the already-low estimates in our study would only overestimate the quality of care as a result of Hawthorne effects. Exploration of how other widely available databases (eg, health-information management systems, Service Delivery Indicators, Demographic and Health Surveys) could be used to measure the quality of primary care could help to build the evidence for a global data architecture for primary care quality. Since the 2007 World Bank publication^32^ on tools and techniques for measurement of service delivery in health, changes have been made in facility surveys, such as the introduction of additional measures of patient experience, but more needs to be done. We outline domains for which better indicators need to be created and set globally to enhance monitoring of the quality of primary care service delivery in LMICs.

Furthermore, our findings could be useful for policy making in several ways. First, the measurement gaps identified in SPAs could be used to inform design of future facility surveys that would provide comprehensive quality-of-care assessments. The availability of quality-focused data collection instruments would allow for policies to be directed towards achievement of better performance scores across all domains of the HQSS framework. Second, although our summary scores for the quality of primary care processes should be cautiously interpreted in view of all the limitations, our findings suggest that quality remains poor across LMICs despite many years of enhancing capacity for strengthening of primary care systems. Such findings lead to questions about more effective resource allocation to improve primary care quality. For example, the lowest scores in most countries were in user experience measures, which suggests a need for policies directed towards enhancing patient–provider relations, and patient experience in general through improvement of health literacy and empowering of both actors (patients and health professionals) on quality of care.

At this stage, we require data beyond what is available from SPAs, because existing data sources comprise mostly descriptive data and are only partial measures of quality. However, our findings can be used to identify areas of poor and good performance and to decide what to prioritise. Future research should assess whether these indices or measures are associated with outcomes.

In summary, we found that even the most comprehensive standardised facility surveys available are of limited use for the measurement of the quality of primary care in LMICs. However, these partial measures showed that quality of primary care is poor across countries and variable within countries 40 years after the Alma-Ata Declaration. On the basis of these incomplete data, primary care does not seem to be well positioned to deliver the Sustainable Development Goals, nor is it likely that people will select primary care over the growing range of other care options for their health needs. The movement for universal health coverage should put system-wide improvement in quality on par with financing reforms.

## Footnotes

* Calculated as continuous indicators (rather than as a binary 0=no, yes=1).

## Footnotes

* Calculated as continuous indicators (rather than as a binary 0=no, yes=1).

* Calculated as continuous indicators (rather than as a binary 0=no, yes=1).

* Calculated as continuous indicators (rather than as a binary 0=no, yes=1).

* Calculated as continuous indicators (rather than as a binary 0=no, yes=1).

* Calculated as continuous indicators (rather than as a binary 0=no, yes=1).

* Calculated as continuous indicators (rather than as a binary 0=no, yes=1).

* Calculated as continuous indicators (rather than as a binary 0=no, yes=1).

* Calculated as continuous indicators (rather than as a binary 0=no, yes=1).

* Calculated as continuous indicators (rather than as a binary 0=no, yes=1).

* Calculated as continuous indicators (rather than as a binary 0=no, yes=1).
