# Supplementary material for: Assessment of quality of primary care with facility surveys: a descriptive analysis in ten low-income and middle-income countries
Source: Lancet Glob Health. 2018 Oct 12;6(11):e1176–85. doi: 10.1016/S2214-109X(18)30440-6 (PMC6187280; doi:10.1016/S2214-109X(18)30440-6)
Supplement: Supplementary appendix [file mmc1.pdf]

THE LANCET
Global Health

Supplementary appendix

This appendix formed part of the original submission and has been peer reviewed. We post it as supplied by the authors.

Supplement to: Macarayan EK, Gage AD, Doubova SV, et al. Assessment of quality of primary care with facility surveys: a descriptive analysis in ten low-income and middle-income countries. *Lancet Glob Health* 2018; **6:** e1176–85.

Supplementary Appendix

Assessment of quality of primary care with facility surveys: a descriptive analysis in ten low- and middle-income countries

List of abbreviations

DHS	Demographic Health Surveys
HQSS	The Lancet Commission on High Quality Health Systems in the SDG Era
HMIS	Health management information systems
LMICs	Low- and middle-income countries
PHC	Primary health care
SARA	Service Availability and Readiness Assessment
SI	Supplementary information
SPA	Service Provision Assessment

Appendix 1: Evidence Before the Study

We conducted a scoping review from August 10 2017 to December 10 2017 to examine evidence quality of primary care. In view of the broad scope of this inquiry, we followed published guidance on scoping reviews. This involved use of a broad search strategy to identify relevant studies; selection of studies according to inclusion and exclusion criteria; charting the data; collating, summarizing, and reporting the results; and placing particular emphasis on consultations with relevant experts.

Database search strategy and selection criteria. We searched PubMed, Embase, and Web of Science using predefined search terms (**Appendix Table 1**) in regards to quality of primary care systems. Only English, peer-reviewed journal articles published since 2000 were included, therefore letters, editorials, conference proceedings, and grey literature, such as reports from government agencies, were excluded in the study. Abstracts and titles of articles are reviewed first in regards to our inclusion and exclusion criteria (**Appendix Table 2**). Then, full-text articles were reviewed. Using Covidence, a second reviewer blinded to the primary reviewer's decisions checked the article selection and data extraction. Differences of opinion were discussed, and a third reviewer resolved any conflicts.

Survey search strategy. We also reviewed survey datasets on quality of primary care. These surveys included: Demographic Health Surveys (DHS), the Service Provision Assessment Surveys (SPA), the UNICEF Multiple Indicator Clusters Survey (MICS), the World Bank's Service Delivery Indicators Surveys, the WHO STEPwise approach to surveillance (STEPS) surveys, and the InterAmerican Development Bank Specialized Datasets, among others.

Appendix Table 1.

Database	Search terms	Filters:
PubMed<br><br>N = 7097	("Quality of Health Care"[mh:noexp] OR "Quality of Care"[tiab] OR "healthcare quality"[tiab] OR "care quality"[tiab] OR "quality of healthcare"[tiab] OR "quality of health care"[tiab] OR "quality of care"[tiab] OR "Quality Indicators, Health Care"[mh] OR health quality indicators[tiab] OR (healthcare quality indicator[tiab] OR healthcare quality indicators[tiab]) OR (care quality indicator[tiab] OR care quality indicators[tiab]) OR "Global trigger tool"[tiab] OR healthcare quality metrics[tiab] OR care quality metrics[tiab] OR (health quality measurement[tiab] OR health quality measures[tiab]) OR (healthcare quality measurement[tiab] OR healthcare quality measures[tiab]) OR (care quality measure[tiab] OR care quality measurement[tiab] OR care quality measures[tiab]) OR "Process Assessment (Health Care)"[Mh] OR "Evidence-Based Practice"[Mh:noexp] OR "Evidence-Based Medicine"[Mh] OR "Evidence-Based Nursing"[Mh] OR "Clinical Competence"[Mh] OR "clinical competency"[tiab] OR "clinical competencies"[tiab] OR "clinical skill"[tiab] OR "clinical skills"[tiab] OR "patient safety"[Mh] OR "patient safety"[tiab] OR "patient harm"[Mh] OR "patient harm"[tiab] OR "Delivery of Health Care, Integrated"[Mh] OR "Integrated Delivery of Health Care"[tiab] OR "Integrated Delivery of Healthcare"[tiab] OR "Integrated Health Care Systems"[tiab] OR "Integrated Healthcare Systems"[tiab] OR "integrated care systems"[tiab] OR "Patient Care Planning"[Mh] OR "continuum of care"[tiab] OR "care continuum"[tiab] OR "continuity of care"[tiab] OR "care continuity"[tiab] OR "care performance"[tiab] OR "Quality Assurance, Health Care"[Mh:noexp] OR "healthcare quality assurance"[tiab] OR "care quality assurance"[tiab] OR (care quality assessment[tiab] OR care quality assessments[tiab]) OR (healthcare quality assessment[tiab] OR healthcare quality assessments[tiab]) OR "Guideline Adherence"[Mh] OR "Guideline	("loattrfull text"[sb] AND ("2000/01/01"[PDAT] : "3000/12/31"[PDAT]) AND "humans"[Mh] AND English[lang])

	Adherence"[tiab] OR "Protocol Compliance"[tiab] OR "Standard of Care"[Mh] OR "Standards of Care"[tiab] OR "Care Standardization"[tiab] OR "Patient Satisfaction"[Mh] OR "Patient Satisfaction"[tiab] OR (patient preference[tiab] OR patient preferences[tiab]) OR (patient experience[tiab] OR patient experienced[tiab] OR patient experiences[tiab]) OR "patient-centered care"[Mh] OR "Patient centered care"[tiab] OR "Patient centred care"[All Fields] OR "patient focused care"[tiab] OR "patient centered nursing"[tiab] OR "professional-patient relations"[Mh] OR (professional patient relation[tiab] OR professional patient relations[tiab] OR professional patient relationship[tiab] OR professional patient relationships[tiab]) OR (nurse patient relation[tiab] OR nurse patient relations[tiab] OR nurse patient relationship[tiab] OR nurse patient relationships[tiab]) OR (doctor patient relation[tiab] OR doctor patient relations[tiab] OR doctor patient relationship[tiab] OR doctor patient relationships[tiab]) OR (physician patient relation[tiab] OR physician patient relations[tiab] OR physician patient relationship[tiab] OR physician patient relationships[tiab]) OR (clinician patient relationship[tiab] OR clinician patient relationships[tiab]) OR provider attitude[tiab] OR "care efficacy"[tiab] OR "effectiveness of Care"[tiab] OR "healthcare effectiveness"[tiab] OR "care effectiveness"[tiab] OR "Structure Process Outcome"[tiab] OR "Donabedian model"[tiab] OR "quality improvement"[Mh] OR ("Quality Improvement*"[tiab]	
Embase, Medline, and Embase classics<br><br>N = 141	'health care quality'/mj OR 'health care concepts'/mj OR 'health care facilities and services'/mj OR 'health service'/mj OR 'quality of nursing care'/mj OR health*quality:ab OR 'quality of health':ab,ti OR 'health care quality indicators':ab,ti OR 'health* quality indicators':ab,ti OR 'global trigger tool':ab,ti OR 'health quality metric':ab,ti OR 'health quality measure*':ab,ti OR 'health care process assessment*':ab,ti OR 'evidence based practice':ab,ti OR 'evidence based medicine':ab,ti OR 'evidence based nursing':ab,ti OR 'clinical competenc*':ab,ti OR 'clinical skill':ab,ti OR 'patient safety':ab,ti OR 'integrated health* system*':ab,ti OR 'integrated care*':ab,ti OR 'care continu*':ab,ti OR 'continuity of care':ab,ti OR 'care perform*':ab,ti OR 'performance of health*':ab,ti OR 'health care quality assurance*':ab,ti OR 'health* quality assurance':ab,ti OR 'health care quality assessment':ab,ti OR 'health* quality assess*':ab,ti OR 'health* guideline*':ab,ti OR 'protocol compliance':ab,ti OR 'standard of care':ab,ti OR 'care standard*':ab,ti OR 'patient satisfaction':ab,ti OR 'patient preference':ab,ti OR 'patient*care':ab,ti OR 'integrated care':ab,ti OR 'nurse patient relationship':ab,ti OR 'doctor patient relation':ab,ti OR 'provider attitude':ab,ti OR 'efficacy of health':ab,ti OR 'health* efficacy':ab,ti OR 'health effectiveness':ab,ti OR 'effectiveness of health*':ab,ti OR 'care efficacy':ab,ti OR 'effectiveness of care':ab,ti OR 'structure process outcome':ab,ti OR 'donabedian model':ab,ti OR 'total quality management':ab,ti	
Web of Science**<br><br>N = 8,617	TS=(Health care quality access and evaluation) OR TS=(Health services administration) OR TS=(Delivery of health care) OR TS=(Quality of Health Care) OR TS=(Quality of Care) OR TS=(healthcare quality) OR TS=(care quality) OR TS=(quality of healthcare) OR TS=(quality of health care) OR TS=(quality of care) OR TS=(Quality Indicators Health Care) OR TS=(health quality indicators) OR TS=(healthcare quality indicator) OR TS=(healthcare quality indicators) OR TS=(care quality indicator) OR TS=(care quality indicators) OR TS=(Global trigger tool) OR TS=(healthcare quality metrics) OR TS=(care quality metrics) OR TS=(health quality measurement) OR TS=(health quality measures) OR TS=(healthcare quality measurement) OR TS=(healthcare quality	

	measures) OR TS=(care quality measure) OR TS=(care quality measurement) OR TS=(care quality measures) OR TS=(Process Assessment Health Care) OR TS=(Evidence-Based Practice) OR TS=(Evidence-Based Medicine) OR TS=(Evidence-Based Nursing) OR TS=(Clinical Competence) OR TS=(clinical competency) OR TS=(clinical competencies) OR TS=(clinical skill) OR TS=(clinical skills) OR TS=(patient safety) OR TS=(patient safety) OR TS=(patient harm) OR TS=(patient harm) OR TS=(Delivery of Health Care Integrated) OR TS=(Integrated Delivery of Health Care) OR TS=(Integrated Delivery of Healthcare) OR TS=(Integrated Health Care Systems) OR TS=(Integrated Healthcare Systems) OR TS=(integrated care systems) OR TS=(Patient Care Planning) OR TS=(continuum of care) OR TS=(care continuum) OR TS=(continuity of care) OR TS=(care continuity) OR TS=(care performance) OR TS=(Quality Assurance Health Care) OR TS=(healthcare quality assurance) OR TS=(care quality assurance) OR TS=(care quality assessment) OR TS=(care quality assessments) OR TS=(healthcare quality assessment) OR TS=(healthcare quality assessments) OR TS=(Guideline Adherence) OR TS=(Guideline Adherence) OR TS=(Protocol Compliance) OR TS=(Standard of Care) OR TS=(Standards of Care) OR TS=(Care Standardization) OR TS=(Patient Satisfaction) OR TS=(Patient Satisfaction) OR TS=(patient preference) OR TS=(patient preferences) OR TS=(patient experience) OR TS=(patient experienced) OR TS=(patient experiences) OR TS=(patient-centered care) OR TS=(Patient centered care) OR TS=(Patient centred care) OR TS=(patient focused care) OR TS=(patient centered nursing) OR TS=(professional-patient relations) OR TS=(professional patient relation) OR TS=(professional patient relations) OR TS=(professional patient relationship) OR TS=(professional patient relationships) OR TS=(nurse patient relation) OR TS=(nurse patient relations) OR TS=(nurse patient relationship) OR TS=(nurse patient relationships) OR TS=(doctor) OR TS=(patient relation) OR TS=(doctor) OR TS=(patient relations) OR TS=(doctor) OR TS=(patient relationship) OR TS=(doctor) OR TS=(patient relationships) OR TS=(physician patient relation) OR TS=(physician patient relations) OR TS=(physician patient relationship) OR TS=(physician patient relationships) OR TS=(clinician patient relationship) OR TS=(clinician patient relationships) OR TS=(provider attitude) OR TS=(care efficacy) OR TS=(effectiveness of Care) OR TS=(healthcare effectiveness) OR TS=(care effectiveness) OR TS=(Structure Process Outcome) OR TS=(Donabedian model) OR TS=(quality improvement) OR TS=(Quality Improvement*)	

Appendix Table 2. Inclusion and exclusion criteria

**Question**	**Yes**	**No**
1. Is it English-only?		
2. Is it peer reviewed?		
3. Is it published after the year 2000?		
4. Does it focus in a low- and middle-income country?		
5. Were there indicators used to assess quality of primary care?		
7. Is it focusing on refugee or humanitarian contexts or other highly specialized settings such as military services? If yes, exclude these articles.		
8. Does it focus on quality of health care or health service delivery based on the definition of quality in the HQSS framework?		

Other areas for exclusion:

1. The study is focusing on assessment of pharmaceutical products, surgical interventions, diagnostic tools or vaccines for roll-out
2. The paper is a viewpoint, protocol for a study, call for papers, editorials, opinion paper, correspondence, letter, or news article.
3. The paper focuses on prospective studies, proposals, simulations, or mathematical modeling of potential effects of a program/intervention
4. The paper focuses on experience of providers instead of patients or clients of a service/program
5. The paper focuses on improvements on information technology infrastructure (e.g. communications systems, databases, or surveillance systems…. the information use intended for health service delivery
7. The paper focuses on conflict-affected states, including focusing on refugees or for humanitarian contexts
8. The paper focuses on determinants of health outcomes (e.g. quality of life) without assessment of any existing programs implemented to improve quality of health care services; thus, focuses on quality of life instead of quality of care
9. The paper focuses on developing approaches or solutions for considerations in the future
10. The paper focuses on assessing the burden of a disease/issue/problem without assessing an ongoing or improved quality-related program/service/reform
11. The paper focuses on highly specialized settings such as military health facilities

Appendix 2: The High Quality Health Systems Framework
Driven by the values of putting people first, equity, efficiency, and resilience, a high quality health system optimizes health in a given context by consistently delivering care that improves or maintains health, being valued and trusted by all people, responding to changing population needs.

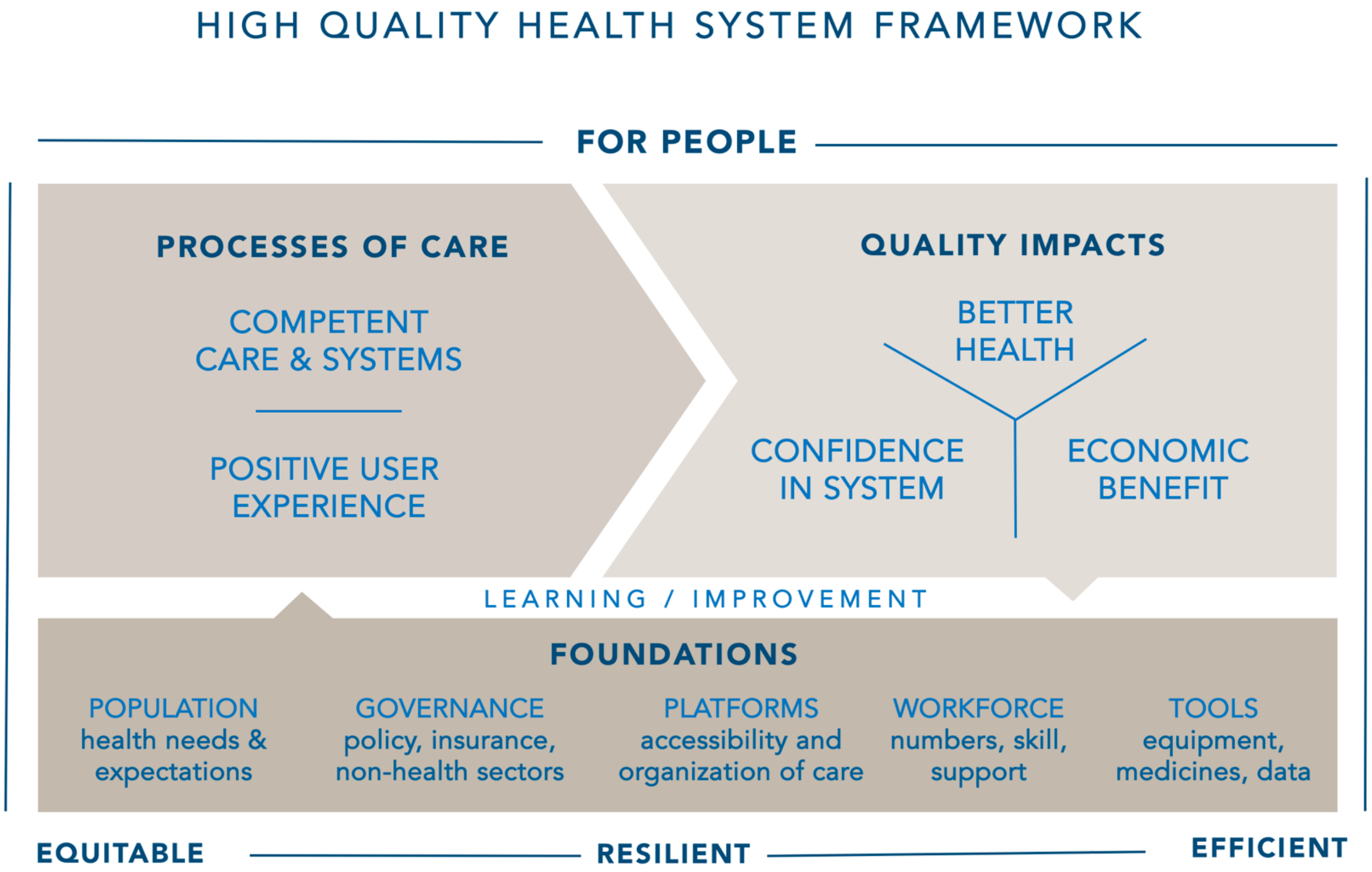

Source: Margaret E. Kruk, Anna D. Gage, Catherine Arsenault, Keely Jordan, Hannah H. Leslie, Sanam Roder-DeWan, Olusoji Adeyi, Pierre Barker, Bernadette Daelmans, Svetlana Doubova, Michael English, Margaret Gakuo Kenyatta, Ezequiel García Elorrio, Vuyokazi Gonyela, Frederico Guanais, Oye Gureje, Lisa R. Hirschhorn, Lixin Jiang, Edward Kelley, Ephrem Tekle Lemango, Jerker Liljestrand, Address Malata, Tanya J. Marchant, Malebona Precious Matsoso, John G. Meara, Nila Moeloek, Manoj Mohanan, Youssoupha Ndiaye, Ole F. Norheim, K. Srinath Reddy, Alexander K. Rowe, Joshua A. Salomon, Gagan Thapa, Nana

A. Y. Twum-Danso, Muhammad Pate High quality health systems—time for a revolution: Report of the Lancet Global Health Commission on High Quality Health Systems in the SDG Era. 2018-forthcoming.

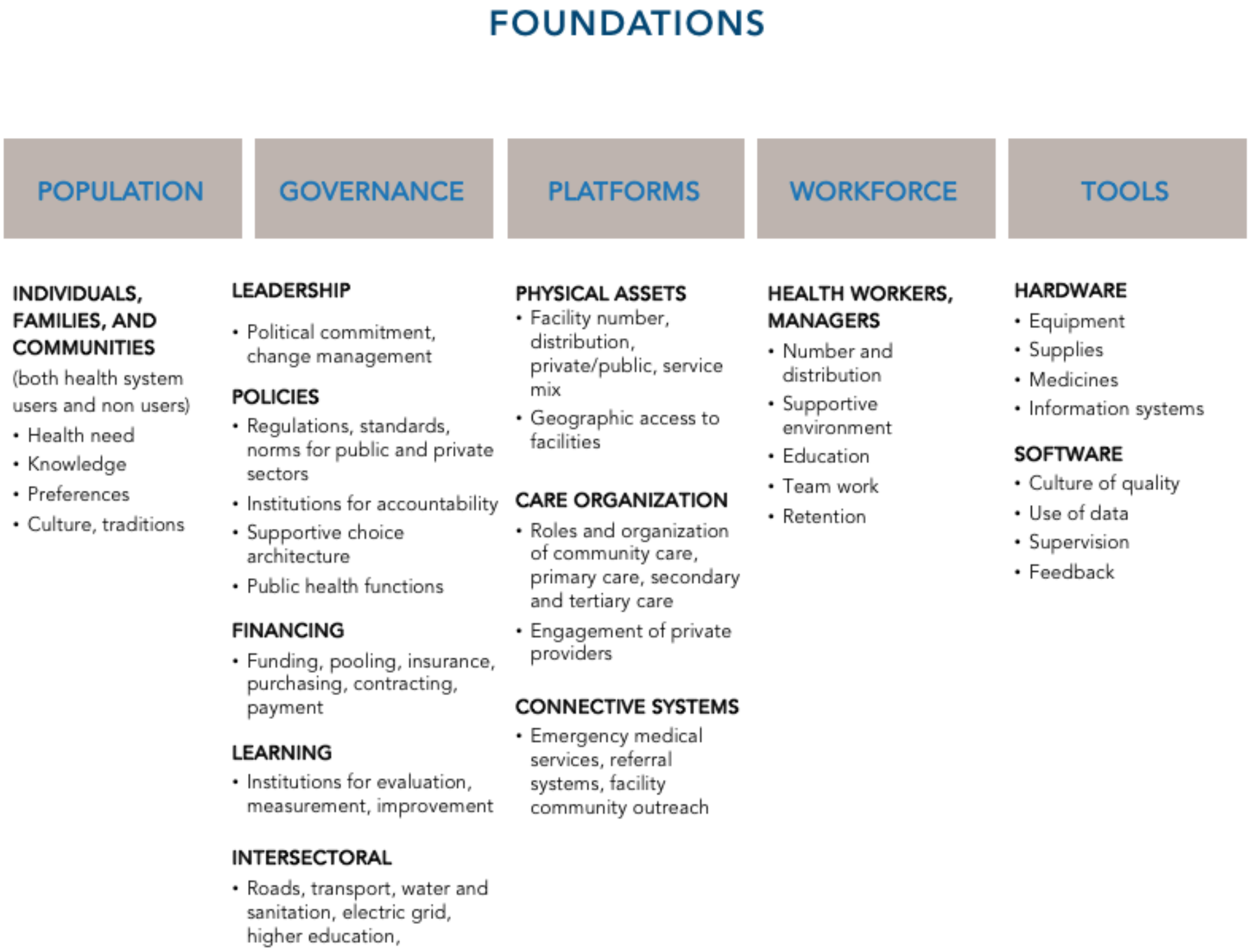

Source: Margaret E. Kruk, Anna D. Gage, Catherine Arsenault, Keely Jordan, Hannah H. Leslie, Sanam Roder-DeWan, Olusoji Adeyi, Pierre Barker, Bernadette Daelmans, Svetlana Doubova, Michael English, Margaret Gakuo Kenyatta, Ezequiel García Elorrio, Vuyokazi Gonyela, Frederico Guanais, Oye Gureje, Lisa R. Hirschhorn, Lixin Jiang, Edward Kelley, Ephrem Tekle Lemango, Jerker Liljestrand, Address Malata, Tanya J. Marchant, Malebona Precious Matsoso, John G. Meara, Nila Moeloek, Manoj Mohanan, Youssoupha Ndiaye, Ole F. Norheim, K. Srinath Reddy, Alexander K. Rowe, Joshua A. Salomon, Gagan Thapa, Nana

A. Y. Twum-Danso, Muhammad Pate High quality health systems—time for a revolution: Report of the Lancet Global Health Commission on High Quality Health Systems in the SDG Era. 2018-forthcoming

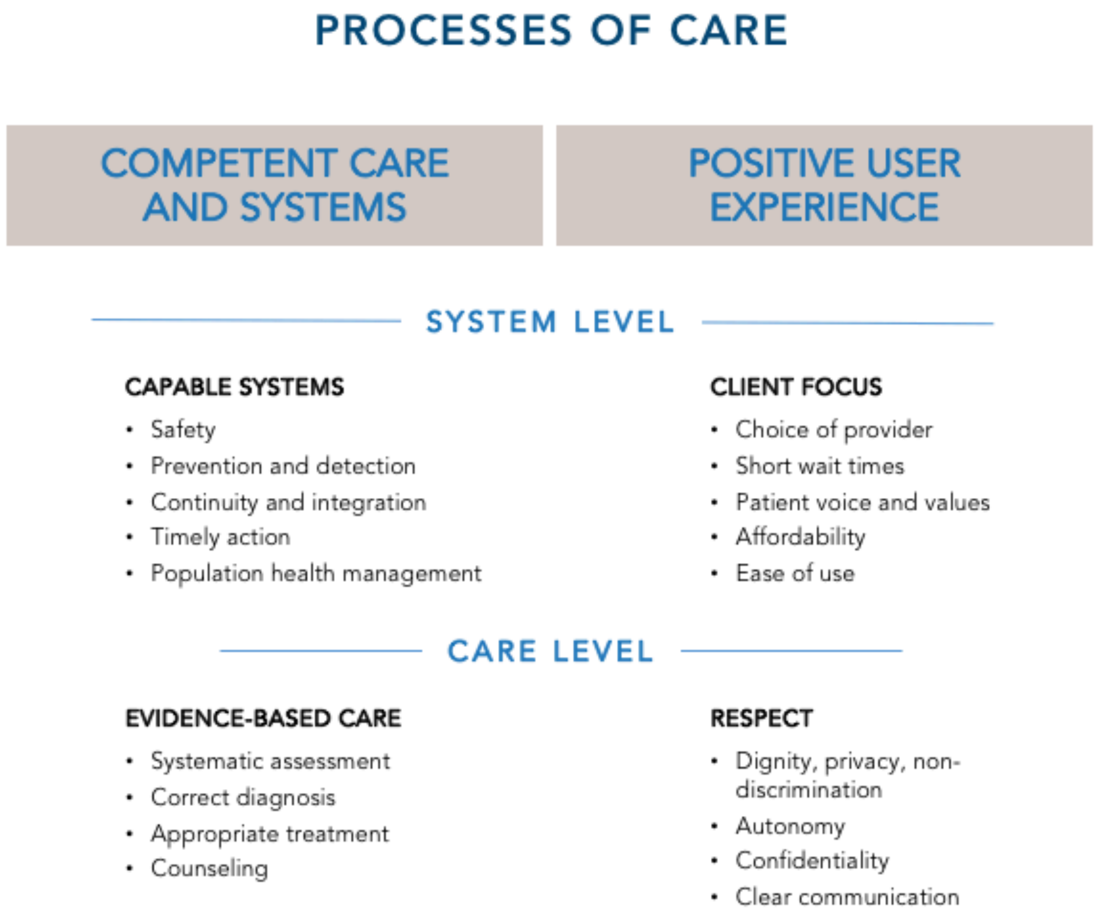

Source: Margaret E. Kruk, Anna D. Gage, Catherine Arsenault, Keely Jordan, Hannah H. Leslie, Sanam Roder-DeWan, Olusoji Adeyi, Pierre Barker, Bernadette Daelmans, Svetlana Doubova, Michael English, Margaret Gakuo Kenyatta, Ezequiel García Elorrio, Vuyokazi Gonyela, Frederico Guanais, Oye Gureje, Lisa R. Hirschhorn, Lixin Jiang, Edward Kelley, Ephrem Tekle Lemango, Jerker Liljestrand, Address Malata, Tanya J. Marchant, Malebona Precious Matsoso, John G. Meara, Nila Moeloek, Manoj Mohanan, Youssoupha Ndiaye, Ole F. Norheim, K. Srinath Reddy, Alexander K. Rowe, Joshua A. Salomon, Gagan Thapa, Nana A. Y. Twum-Danso, Muhammad Pate High quality health systems—time for a revolution: Report of the Lancet Global Health Commission on High Quality Health Systems in the SDG Era. 2018-forthcoming

QUALITY IMPACTS

BETTER HEALTH

Level and distribution of:

- Clinical outcomes (mortality, morbidity)
- Patient reported outcomes (function, symptoms, pain, wellbeing, quality of life, avoiding serious health-related suffering)

CONFIDENCE IN SYSTEM

- Satisfaction
- Recommendation
- Trust
- Care uptake and retention

ECONOMIC BENEFIT

- Financial risk protection
- Ability to work or attend school
- Economic growth

Source: Margaret E. Kruk, Anna D. Gage, Catherine Arsenault, Keely Jordan, Hannah H. Leslie, Sanam Roder-DeWan, Olusoji Adeyi, Pierre Barker, Bernadette Daelmans, Svetlana Doubova, Michael English, Margaret Gakuo Kenyatta, Ezequiel García Elorrio, Vuyokazi Gonyela, Frederico Guanais, Oye Gureje, Lisa R. Hirschhorn, Lixin Jiang, Edward Kelley, Ephrem Tekle Lemango, Jerker Liljestrand, Address Malata, Tanya J. Marchant, Malebona Precious Matsoso, John G. Meara, Nila Moeloek, Manoj Mohanan, Youssoupha Ndiaye, Ole F. Norheim, K. Srinath Reddy, Alexander K. Rowe, Joshua A. Salomon, Gagan Thapa, Nana A. Y. Twum-Danso, Muhammad Pate High quality health systems—time for a revolution: Report of the Lancet Global Health Commission on High Quality Health Systems in the SDG Era. 2018-forthcoming

Appendix 3: STROBE Checklist

	Item No	Recommendation	Page Number
**Title and abstract**	1	(*a*) Indicate the study's design with a commonly used term in the title or the abstract	1
		(*b*) Provide in the abstract an informative and balanced summary of what was done and what was found	2
**Introduction**			
Background/rationale	2	Explain the scientific background and rationale for the investigation being reported	5
Objectives	3	State specific objectives, including any pre-specified hypotheses	5
**Methods**			
Study design	4	Present key elements of study design early in the paper	6
Setting	5	Describe the setting, locations, and relevant dates, including periods of recruitment, exposure, follow-up, and data collection	6-7
Participants	6	(*a*) Give the eligibility criteria, and the sources and methods of selection of participants	6-7
Variables	7	Clearly define all outcomes, exposures, predictors, potential confounders, and effect modifiers. Give diagnostic criteria, if applicable	6-8
Data sources/ measurement	8*	For each variable of interest, give sources of data and details of methods of assessment (measurement). Describe comparability of assessment methods if there is more than one group	6-8
Bias	9	Describe any efforts to address potential sources of bias	7-8
Study size	10	Explain how the study size was arrived at	7-8
Quantitative variables	11	Explain how quantitative variables were handled in the analyses. If applicable, describe which groupings were chosen and why	7-8
Statistical methods	12	(*a*) Describe all statistical methods, including those used to control for confounding	7-8
		(*b*) Describe any methods used to examine subgroups and interactions	7-8
		(*c*) Explain how missing data were addressed	7-8
		(*d*) If applicable, describe analytical methods taking account of sampling strategy	7-8
		(*e*) Describe any sensitivity analyses	
**Results**			
Participants	13*	(a) Report numbers of individuals at each stage of study—eg numbers potentially eligible, examined for eligibility, confirmed eligible, included in the study, completing follow-up, and analysed	9
		(b) Give reasons for non-participation at each stage	
		(c) Consider use of a flow diagram	
Descriptive data	14*	(a) Give characteristics of study participants (eg demographic, clinical, social) and information on exposures and potential confounders	9
		(b) Indicate number of participants with missing data for each variable of interest	Page 9, Table 2, Supplementary Information 5
Outcome data	15*	Report numbers of outcome events or summary measures	Page 9, Table 2, Supplementary Information 5
Main results	16	(*a*) Give unadjusted estimates and, if applicable, confounder-adjusted estimates and their precision (eg, 95% confidence interval). Make clear which confounders were adjusted for and why they were included	
		(*b*) Report category boundaries when continuous variables were categorized	Page 9, Table 2, Supplementary Information 5
		(*c*) If relevant, consider translating estimates of relative risk into absolute risk for a meaningful time period	
Other analyses	17	Report other analyses done—eg analyses of subgroups and interactions, and sensitivity analyses	Page 9, Table 2, Supplementary Information 5
**Discussion**			
Key results	18	Summarise key results with reference to study objectives	9-11
Limitations	19	Discuss limitations of the study, taking into account sources of potential bias or imprecision. Discuss both direction and magnitude of any potential bias	12-13

Interpretation	20	Give a cautious overall interpretation of results considering objectives, limitations, multiplicity of analyses, results from similar studies, and other relevant evidence	12-13
Generalisability	21	Discuss the generalisability (external validity) of the study results	11-13
**Other information**			
Funding	22	Give the source of funding and the role of the funders for the present study and, if applicable, for the original study on which the present article is based	8

Appendix 4: Descriptions for High-Quality Health Systems Framework domains and subdomains

Domains	Subdomains	Descriptions specific to primary care*
**Competent systems**	a. Safety	Primary care systems seek to prevent harm to patients by ensuring facility cleanliness, safety precautions and other safety interventions (e.g. sterilization, sharp and waste disposal, infection control items), among others. An unsafe primary care system predisposes patients to adverse events and injuries due to medical devices, injuries due to surgical and anesthesia errors including wrong-site surgery, health care-associated infections, improper transfusions and injection practices, falls, burns, and pressure ulcers.
	b. Prevention and detection	The prevention and early detection of diseases, including thorough screening where indicated or referrals when needed, is an important function of high quality health systems especially primary care systems.
	c. Continuity and integration	Continuity of care is reflected by the health system's ability to retain people in care and for the patient, by his or her ability to see a clinician familiar with their medical history. Integration is the extent to which health services are delivered in a complementary and coherent manner. Scheduling follow-up visits and tracking care using vaccination cards and client records are some examples of ensuring continuous and integrated primary care systems.
	d. Population health management	Population health management such as outreach services and community meetings is core to primary care systems, which should collect, analyze, and act upon data on patient population to optimize how to best manage specific diseases within that population.
	e. Timely action	Timely actions in primary care systems optimize patient outcomes and reduce the need for additional admissions due to complications arising from service provision. Timeliness is also central for conditions that can be cured if treated early, including many cancers, and conditions such as tuberculosis or diabetes, where early treatment prevents transmission or disease progression. For people with life-threatening emergencies, such as labor complications, trauma, and strokes, treatment delays substantially increase mortality risk.
**Evidence-based care**	Technical quality indices for the following services:<br>a. Antenatal care<br>b. Family planning<br>c. Sick child care<br><br>See S1B for specific indicators under evidence-based care for each type of service.	Evidence-based care is exhibited when there is systematic assessment, correct diagnosis, appropriate treatment, and counseling.<br>- A systematic patient assessment involves gathering clinically relevant information by asking appropriate history questions and performing recommended examinations and tests.<br>- Incorrect diagnoses have deleterious consequences on health and contribute to treatment delays and antimicrobial resistance.<br>- Treatments should be appropriate. Underuse of effective care and the overuse of unnecessary care lead to poor primary care quality.<br>- Proper counseling and client education are vital elements of evidence-based care. For example, during antenatal care, many skilled providers fail to advise women on the signs of pregnancy complications or how to prevent HIV infections, and when prescribing contraceptives, many fail to discuss their potential side effects.
**User experience**	a. Client focus	Providers have shown care that is respectful of, and responsive to, individual patient preferences, needs, and values.
	b. Clear communication	Clear communication is exhibited when providers have adequately explained and discussed care plans and treatment processes such as follow up visits, use of family planning methods and their side effects or other danger signs.

*Descriptions were based from the report of the Lancet Global Health Commission on High Quality Health Systems in the SDG era.

Appendix 4: Sampling and Survey Weights
Source: The DHS Program - SPA Methodology. https://dhsprogram.com/What-We-Do/Survey-Types/SPA-Methodology.cfm (accessed Sept 30, 2017).

The Service Provision Assessment (SPA) survey is a health facility assessment that provides a comprehensive overview of a country's health service delivery. Typically, SPA surveys collect data from 400-700 facilities, selected from a comprehensive list of health facilities in a country (sampling frame), categorized by facility type, managing authority (public and non-public), and by region. The sample is selected to provide indicators at the national level for the different facility types and managing authority as well as aggregate indicators at the regional level. SPA surveys are typically fielded by 10-15 teams, each comprised of 3-4 interviews mostly health workers. These interviewers collect data from the facility in-charge and the most knowledgeable persons available for each service. Data were weighted by the country SPA to account for differentials caused by oversampling and undersampling and to represent the actual distribution of facilities in the country.

The SPA country reports detail the sampling and survey methodology as follows:

1. Uganda 2007 - Data were collected from a representative sample of facilities in the country, a sample of health service providers at each sampled facility, and a sample of sick children, family planning, ANC, and STI clients. The sample used for the 2007 USPA was obtained from a list of 3,000 functioning health facilities in Uganda at the time of the survey. The list included hospitals and health centres (HC-IVs, HC-IIIs, and HC-IIs) with different managing authorities, including government, private-for-profit, parastatal, and faith-based organisations. For the purposes of the survey, specialised HIV/AIDS facilities or clinics, such as The AIDS Support Organisation (TASO), that may be providing purely HIV services (such as HIV counselling and testing, or antiretroviral therapy only) are categorised with HC-IIs. All facilities not managed by the government (private-for-profit, NGO, and faith-based facilities) were grouped together as private facilities. A sample size of 500 facilities was selected initially for the survey, based on logistic considerations as well as the minimum sample size required for the desired analysis. The sample allows for national and regional estimates for key indicators. All hospitals throughout Uganda (national referral, regional, general, and other hospitals), and about half of all HC-IVs were purposely included in the sample. HC-IIIs and HC-IIs were sampled in such a way as to provide national and regional-level representation. Thus, the USPA final sample covered approximately 16 percent of all facilities in the country. Data were weighted to account for differentials caused by oversampling and undersampling and to represent the actual distribution of facilities in the country. Source: Ministry of Health Kampala Uganda. 2007 Uganda Service Provision Assessment Survey. 2008; published online August 2008. https://www.dhsprogram.com/pubs/pdf/SPA13/SPA13.pdf.

2. Rwanda 2007 - Data were collected from a sample of facilities, a sample of health service providers at each facility, and a sample of caretakers of sick children, and family planning, ANC, and STI clients. The survey visited all public health facilities and a sample of private facilities that include all of those with five or more staff at the time of listing and one-third of the facilities with three to four staff. Private health facilities with one or two staff were not included in the survey. The sample included hospitals, health centers, dispensaries, health posts, polyclinics, and clinics, with different managing authorities, including government, government assisted, nongovernmental organization (NGO), and community. Out of a total of 555 facilities initially selected for the 2007 RSPA, 538 were successfully interviewed. This represents a response rate of 97 percent. The sample includes 42 hospitals (8 percent), 389 health centers and polyclinics (72 percent), and 107 dispensaries, health posts and clinics (20 percent). More than half (57 percent) of the facilities are government facilities, managed mainly by the MOH. Government-assisted1

facilities represent one-fourth of facilities, while private, nongovernmental organization (NGO) and community facilities represent 18 percent of facilities. The distribution of health care facilities in South, East, and West provinces is about the same (21 to 25 percent). About 17 percent of the facilities are in North province and 16 percent are in Kigali City. The sample of health service providers was selected from providers who were present in the facility on the day of the survey and who provided services that were assessed by the 2007 RSPA. Attempts were made to interview an average of eight providers per facility. In facilities with fewer than eight health providers, all of the providers present on the day of the visit were interviewed. In facilities with more than eight providers, an average of eight providers was interviewed, including all providers whose work was observed. If interviewers observed fewer than eight providers, then they also interviewed a random selection of the remaining health providers to obtain an average of eight provider interviews. Source: Rwanda Service Provision Assessment Survey - The DHS Program. https://dhsprogram.com/pubs/pdf/SPA15/SPA15.pdf.

3. Namibia 2009 – Since the total number of facilities in the country is relatively small, the 2009 NHFC visited all facilities. A master list of 446 health facilities in Namibia was obtained from the division of the MoHSS that is responsible for health facility registration in the country. The list included hospitals, health centres, clinics, stand-alone VCT centres, and sick bays; these facilities were under various management authorities, including government, private-for-profit, mission, NGOs, ministry of defence (MoD), and the Namibia police. Small doctor's consultation rooms were not included in the assessment. The sample of health service providers was selected from providers who were present in the facility on the day of the census and who provided services that were assessed by the NHFC. In facilities with fewer than eight health service providers, efforts were made to interview all of the providers present on the day of the visit using the health worker interview questionnaire. In facilities with more than eight providers, efforts were made to interview an average of eight providers, including all providers whose work was observed. If interviewers observed fewer than eight providers, then they also interviewed a random selection of the remaining providers to obtain an average of eight provider interviews. Data were weighted during analysis to account for the differentials caused by over-sampling or under-sampling of providers with a particular qualification in a facility type or region. In a few cases the staff present on the day of the census may not be representative of the staff who normally provide the services being assessed. Source: Ministry of Health and Social Services-MoHSS/Namibia, Macro ICF. Namibia Health Facility Census (HFC) 2009. 2011. http://dhsprogram.com/pubs/pdf/SPA16/SPA16.pdf (accessed Sept 30, 2018).

4. Kenya 2010 – 703 facilities were sampled for the 2010 SPA survey. Hospitals, health centers, maternities, and stand-alone VCT facilities for example were oversampled since they exist in small numbers in the country and also provide most of the maternal health and HIV and AIDS services. The 2010 KSPA collected data from 252 sampled hospitals, which corresponds to about 36 percent of the total sample. However, the true proportion of hospitals in relation to other facility types as per the sampling frame for the 2010 KSPA survey is only 7 percent. Thus, for analysis, the number of hospitals was adjusted down to 51, which approximately reflects the actual percentage (7 percent) of the sample. Source: Ministry of Medical Services Ministry of Public Health and Sanitation. Kenya Service Provision Assessment Survey 2010. 2010. https://dhsprogram.com/pubs/pdf/SPA17/SPA17.pdf.

5. Malawi 2013 – The 2013-14 MSPA was designed to be a census of all formal-sector health facilities in Malawi. The Central Monitoring and Evaluation Division (CMED) of the Malawi MoH provided a master list of 1,060 such facilities. Data were weighted to account for differentials caused by non-response and closure of some facilities. For health providers, the sample was selected from providers who were present in the facility on the day of the assessment

and who provided services that were assessed by the 2013-14 SPA. The aim was to interview an average of eight providers in each facility in order to include providers of the range of services being assessed. In facilities with fewer than eight health care providers, all the providers present on the day of the visit were interviewed. In facilities with more than eight providers, efforts were made to interview eight providers, including all providers whose consultations were observed, and those who provided information for any section of the Facility Inventory questionnaire. If interviewers observed fewer than eight providers, then they also interviewed a random selection of the remaining health care providers to obtain a total of eight provider interviews. Data were weighted during analysis to account for the differentials caused by over-sampling or under-sampling of providers with a particular qualification in a facility type or province. In a few cases the staff members present on the day of the assessment may not have been representative of the staff that usually provides the services being assessed. Source: Ministry of Health Lilongwe, Malawi. Malawi Service Provision Assessment 2013-14. 2013. https://dhsprogram.com/pubs/pdf/SPA20/SPA20%5BOct-7-2015%5D.pdf.

6. Haiti 2013 – The 2013 HSPA is an evaluation of both public and sector health institutions in Haiti. The survey was conducted in all registered health institutions (hospitals, health, and dispensaries) across the country's 10 departments. Administrators and service providers health services from these institutions were interviewed; providers and patients / clients who came consultation for specific health services (consultation of sick children under five, Family Planning and Prenatal Counseling) were observed during the consultations and Interviews were conducted with patients whose consultations had been observed. Before the start of the survey, the MSSP had provided the IHE with a list of institutions that contained no information regarding the address and references of the persons in charge. Before the visit investigators, two IHE coordinators accompanied by field officers went through all the country to make a physical inventory of all health institutions in the country, to obtain a physical address and a contact to facilitate the work of the field teams. Of the 908 institutions MSPP list, 735 were found, 87 were not found and 86 were closed and were not functioning at the time of the survey. In addition, during the enumeration work, both Coordinators identified 172 new institutions that were not on the MSPP list. In total, 907 institutions were assigned to the field teams to be investigated. Source: Institut Haïtien de l'Enfance-IHE, ICF International. Haïti Évaluation de la Prestation des Services de Soins de Santé 2013. 2014. http://dhsprogram.com/pubs/pdf/SPA19/SPA19.pdf (accessed Sept 30, 2018).

7. Ethiopia 2014 – 1,165 functional facilities were sampled for the 2014 SPA survey. Hospitals and health centers were over-sampled since they exist in small numbers in the country and also provide most of the maternal health and HIV and AIDS services. The 2014 SPA collected data from all 233 hospitals, which corresponds to about 18 percent of the total sample. However, true proportion of hospitals in relation to other facility types as per the sampling frame for the 2014 SPA survey is only 1 percent. Thus, for analysis, the number of hospitals were adjusted down to 12, which approximately reflects the actual percentage (1 percent) of the sample. Source: Ethiopia Service Provision Assessment Plus Survey 2014. Demographic Health Surveys.

8. Nepal 2015 - A master list of 4,719 formal-sector health facilities in Nepal was obtained from the MoH and used as the sampling frame for the survey. The majority of the facilities in the sampling frame were health posts (80 percent). For private hospitals, only those having 15 beds or more were included in the master list. A total of 1,000 facilities were selected for the survey. By design, the sample included all nonspecialized government hospitals, all private hospitals with 100 or more inpatient beds, and all PHCCs. The remainder of the sample consisted of sampled health posts, private hospitals with at least 15 beds but fewer than 100 beds, stand-alone HTC sites, and UHCs. Eight sampled facilities turned out to be duplicates, resulting in an effective

sample size of 992 facilities. The sample of health service providers was selected from providers who were present in the facility on the day of the assessment and who provided services assessed in the 2015 NHFS. The aim was to interview an average of eight providers in each facility in order to include providers of the range of services being assessed. In facilities with fewer than eight health care providers, all of the providers present on the day of the visit were interviewed. In facilities with more than eight providers, efforts were made to interview eight providers, including all providers whose consultations were observed and who responded to any section of the facility inventory questionnaire. If interviewers observed fewer than eight providers, then they also interviewed a random selection of the remaining providers to obtain a total of eight provider interviews. Data were weighted during the analysis to account for the differentials caused by oversampling or undersampling of providers with a particular qualification in a facility type or province. In a few cases, the staff members present on the day of the assessment may not have been representative of the staff usually providing the services being assessed. Source: The DHS Program - Nepal: SPA, 2015. https://dhsprogram.com/what-we-do/survey/survey-display-400.cfm (accessed Sept 30, 2018).

9. Senegal 2015-16 - As in previous phases, SSPA is a survey of health structures in the sector than the private sector in Senegal. The survey was conducted in all identified health facilities (hospitals, health centers, and health posts), as well as health huts related to health posts selected in the 14 regions of the country. Administrators and health service providers of these structures were interviewed; providers and patients / clients who come for consultation for services health care (consultation of the sick child under five and family planning) have been observed during the consultations and interviews were made accompanying persons with sick children whose consultations had been observed. Out of a total of 3,764 health facilities (including 68 hospitals, 148 health centers, 1,853 health and 1,695 health huts), the sample included 54% of hospitals (37), 51% of health centers (75) including garrison medical centers (CMG), 16% of health posts (302) including infirmaries prisons and correctional facilities (MAC) and 23% of health huts (390). Source: de la Démographie-ANSD/Sénégal AN de la S et, de la Santé et de l'Action Sociale M, ICF. Senegal: Enquête Continue sur la Prestation des Services de Soins de Santé (ECPSS) 2017. 2018. http://dhsprogram.com/pubs/pdf/SPA27/SPA27.pdf (accessed Sept 30, 2018).

10. Tanzania 2015 - The 2014-15 TSPA was designed to be a sample survey of all formal-sector health facilities in Tanzania. A master list of health facilities that consisted of 7,102 verified (active) health facilities in Tanzania was obtained from the Ministry of Health and Social Welfare (MoHSW) on the Tanzania Mainland and the Ministry of Health (MOH) in Zanzibar. The list included hospitals, health centres, dispensaries, and clinics. These facilities were managed by the government, private-for-profit, parastatal, and faith-based entities. A sample of 1,200 facilities was selected to participate in the survey. The sample was designed to provide nationally representative results by facility type and managing authority and regionally representative results for the 25 Tanzania Mainland regions and the 5 Zanzibar regions (a total of 30 survey regions). For health providers, the aim was to interview an average of eight providers in each facility in order to include providers of the range of services being assessed. In facilities with fewer than eight health care providers, all of the providers present on the day of the visit were interviewed. In facilities with more than eight providers, efforts were made to interview eight providers, including all providers whose consultations were observed, and those who provided information for any section of the Facility Inventory questionnaire. If interviewers observed fewer than eight providers, then they also interviewed a random selection of the remaining health care providers to obtain a total of eight provider interviews. Data were weighted during analysis to account for the differentials caused by oversampling or under-sampling of providers with a particular qualification in a facility type or region. For ANC, family planning, and curative care for sick

children, clients were identified and systematically selected for observation based on the number of clients present at each service site on the day of the visit. Where many clients were present and eligible for observation, the rule was to observe a maximum of five clients for each provider of the service, with a maximum of 15 observations for each service in any given facility. Interviewers attempted to conduct exit interviews with all observed clients or caretakers of observed sick children before they left the facility. When several eligible ANC or family planning clients were waiting, interviewers tried to select two new clients for every follow-up client. The day's caseload and the logistics of organising observations did not always allow them to meet this objective. For child health consultations, only children younger than five years of age who presented with an illness (rather than an injury or a skin or eye infection exclusively) were selected for observation. Seven sampled facilities refused to be surveyed, 4 had closed down, and one facility could not be reached. The remaining 1,188 facilities were successfully interviewed, with a response rate of 99 percent. Among the surveyed facilities, 256 were hospitals, 379 were health centres, 493 were dispensaries, and 60 were clinics. Source: Tanzania Service Provision Assessment Survey 2014-2015 [SPA22]. https://dhsprogram.com/pubs/pdf/spa22/spa22.pdf.

Appendix 5: Components of evidence-based care for antenatal care, family planning, and sick child care

Evidence-based care	Indicators from the Service Provision Assessment Survey
**1. Antenatal care (18)**	**Systematic Assessment (8)**<br>1. Provider asked about any danger signs<br>2. Provider asked any pregnancy history questions<br>3. Provider asked date of start of last menstruation<br>4. Provider measured weight<br>5. Provider palpated abdomen for uterine height<br>6. Provider checked legs/hands/feet for edema<br>7. Provider conducted vaginal exam<br>8. Provider checked blood pressure<br>**Treatment (4)**<br>9. Provider performed or referred for anemia test<br>10. Provider performed or referred for blood group-typing<br>11. Provider prescribed or gave tetanus toxoid injection<br>12. Provider gave or prescribed iron/folic acid this visit<br>**Counseling (6)**<br>13. Provider discussed nutrition<br>14. Provider counseled on use of ITN<br>15. Provider counseled on birth planning - preparation and location<br>16. Provider counseled regarding supplies for delivery<br>17. Provider conducted/looked at/referred patient to ultrasound<br>18. Provider wrote on client card
**2. Family planning (33)**	**Systematic Assessment (18)**<br>1. Provider counseled on postnatal family planning<br>2. Provider asked age<br>3. Provider asked number of living children<br>4. Provider asked last delivery date<br>5. Provider asked last menstrual period<br>6. Provider asked reproductive intentions<br>7. Provider asked desired timing of next child<br>8. Provider assessed current breastfeeding<br>9. Provider assessed regularity of menstruation<br>10. Provider assessed smoking history<br>11. Provider asked STI symptoms<br>12. Provider asked chronic illness history<br>13. Provider checked blood pressure<br>14. Provider weighed client<br>15. Provider conducted pelvic exam<br>16. Provider assessed smoking history<br>17. Provider asked STI symptoms<br>18. Provider asked chronic illness history<br>**Treatment (3)**<br>19. At least one FP method prescribed<br>20. Provider ensured visual privacy<br>21. Provider ensured auditory privacy<br>**Counseling (12)**<br>22. Provider counseled on more than one issue, on more than one method<br>23. Provider assured client of confidentiality<br>24. Provider discussed partner's views on FP<br>25. Provider discussed partner/relationship status<br>26. Provider discussed STI / HIV risk<br>27. Provider discussed STI prevention with condoms

		28. Provider discussed dual method use<br>29. Provider asked client regarding questions or concerns on current method<br>30. Provider used visual aids<br>31. Provider checked client health card<br>32. Provider wrote on client health card<br>33. Provider discussed follow-up visit
**3.**	**Sick child (20)**	**Systematic Assessment (16)**<br>1. Provider asked / caretaker mentioned if child unable to drink or breastfeed<br>2. Provider asked / caretaker mentioned cough or difficult breathing<br>3. Provider asked / caretaker mentioned diarrhea<br>4. Provider asked / caretaker mentioned fever<br>5. Provider asked / caretaker mentioned convulsions<br>6. Provider asked about mother's HIV status<br>7. Provider asked / caretaker mentioned ear pain<br>8. Provider counted respiration for 60 seconds<br>9. Provider weighed client<br>10. Provider plotted weight on growth chart<br>11. Provider took temperature<br>12. Provider checked palms / conjunctiva / mouth for pallor<br>13. Provider checked for edema<br>14. Provider asked if child received vitamin A within past 6 months<br>15. Provider asked if child received any deworming medication in last 6 month<br>16. Provider asked caretaker about vomiting<br>**Diagnosis (1)**<br>17. Provider stated diagnosis to caretaker<br>**Treatment (1)**<br>18. Provider recommended food / liquid intake<br>**Counseling (2)**<br>19. Provider explained dosing if medication prescribed<br>20. Provider described more than 1 danger sign requiring return to facility

Source: Margaret E. Kruk, Anna D. Gage, Catherine Arsenault, Keely Jordan, Hannah H. Leslie, Sanam Roder-DeWan, Olusoji Adeyi, Pierre Barker, Bernadette Daelmans, Svetlana Doubova, Michael English, Margaret Gakuo Kenyatta, Ezequiel García Elorrio, Vuyokazi Gonyela, Frederico Guanais, Oye Gureje, Lisa R. Hirschhorn, Lixin Jiang, Edward Kelley, Ephrem Tekle Lemango, Jerker Liljestrand, Address Malata, Tanya J. Marchant, Malebona Precious Matsoso, John G. Meara, Nila Moeloek, Manoj Mohanan, Youssoupha Ndiaye, Ole F. Norheim, K. Srinath Reddy, Alexander K. Rowe, Joshua A. Salomon, Gagan Thapa, Nana A. Y. Twum-Danso, Muhammad Pate High quality health systems—time for a revolution: Report of the Lancet Global Health Commission on High Quality Health Systems in the SDG Era. 2018-forthcoming

Appendix 6: Performance on indicators of primary care quality index in the study countries

		Overall	Ethiopia	Haiti	Kenya	Malawi	Namibia	Nepal	Rwanda	Senegal	Tanzania	Uganda
	**n**	**7049**	**1104**	**786**	**443**	**941**	**366**	**722**	**496**	**882**	**937**	**372**
**Overall quality score**	**6547**	**0.41**	**0.32**	**0.37**	**0.44**	**0.42**	**0.46**	**0.39**	**0.45**	**0.45**	**0.45**	**0.37**
**1. Competent systems**	**6547**	**0.51**	**0.48**	**0.45**	**0.49**	**0.53**	**0.51**	**0.49**	**0.60**	**0.57**	**0.50**	**0.47**
**a. Safety**	**6547**	**0.57**	**0.42**	**0.55**	**0.65**	**0.68**	**0.57**	**0.54**	**0.52**	**0.75**	**0.57**	**0.40**
Cleanliness of the facility	6520	0.77	0.45	0.76	0.89	0.82	0.91	0.76	0.86	0.85	0.75	0.67
Sterilization of equipment	6061	0.83	0.88	0.86	0.73	0.93	0.07	0.98	0.94	0.99	0.98	0.91
Adequate sharp disposal	6314	0.67	0.56	0.61	0.77	0.79	0.89	0.67	0.73	0.93	0.55	0.25
Waste disposal	6483	0.51	0.38	0.42	0.73	0.57	0.77	0.46	0.62	0.52	0.40	0.24
Rooms with infection control items	6438	0.39	0.29	0.37	0.56	0.36	0.65	0.35	0.00	0.76	0.39	0.17
Guideline for standard precautions	6547	0.28	0.10	0.34	0.25	0.66	0.17	0.06	0.07	0.61	0.39	0.17
**b. Prevention and detection**	**4081**	**0.34**	**0.43**	**0.27**	**0.43**	**0.23**	**0.59**	**0.06**	**0.39**	**0.45**	**0.40**	**0.11**
Urine test screening and referrals	2486	0.33	0.35	0.46	0.38	0.01	0.81	0.21	0.20	0.52	0.33	0.03
Syphilis screening and referrals	2487	0.28	0.30	0.46	0.40	0.04	0.49	0.11	0.41	0.28	0.33	0.03
HIV screening and counseling	2486	0.38	0.39	0.27	0.52	0.49	0.47	0.06	0.56	0.28	0.53	0.26
TB screening and counseling for HIV patients	2366	0.48	0.47	0.64		0.27		0.12		0.86	0.50	
Tests TB patients for HIV	1067	0.41	0.63	0.19		0.41		0.04		0.50	0.69	
**c. Continuity and integration**		**0.59**	**0.48**	**0.57**	**0.62**	**0.59**	**0.74**	**0.58**	**0.62**	**0.67**	**0.56**	**0.49**
Offer core primary care services	6547	0.81	0.79	0.82	0.78	0.76	0.87	0.90	0.78	0.86	0.80	0.76
Discussions during follow-up visits	2212	0.83	0.66	0.72	0.94	0.88	0.97	0.61	0.92	0.80	0.81	0.95
Offer common NCD services	4374	0.65	0.21	0.88		0.66		0.58		0.98	0.58	
ANC, FP, SC, STI services with client records	6473	0.60	0.57	0.59	0.70	0.30	0.86	0.73	0.85	0.55	0.52	0.36
Availability of test return agreements	2364	0.53	0.48	0.25		0.79		0.31		0.84	0.53	
Vaccination cards	4241	0.42	0.35	0.18	0.43	0.78	0.71	0.48	0.16	0.38	0.39	0.33
Postnatal family planning counseling	2488	0.17	0.04	0.09	0.22	0.10	0.22	0.02	0.37	0.08	0.33	0.24
**d. Population health management**	**6547**	**0.48**	**0.56**	**0.38**	**0.22**	**0.54**	**0.20**	**0.73**	**0.71**	**0.40**	**0.46**	**0.58**
Outreach services	6547	0.59	0.69	0.56	0.17	0.67	0.20	0.90	0.83	0.62	0.49	0.81
Staff community meetings	5565	0.29	0.31	0.11	0.27	0.20	0.18	0.48	0.58	0.07	0.43	0.27
**2. Evidence based care**	**5084**	**0.41**	**0.38**	**0.34**	**0.48**	**0.36**	**0.53**	**0.30**	**0.43**	**0.44**	**0.40**	**0.49**
**a. ANC**	**2488**	**0.56**	**0.47**	**0.45**	**0.66**	**0.54**	**0.65**	**0.44**	**0.65**	**0.62**	**0.56**	**0.58**
**b. Family planning**	**3475**	**0.40**	**0.33**	**0.30**	**0.46**	**0.38**	**0.46**	**0.24**	**0.55**	**0.39**	**0.42**	**0.50**
**c. Sick child care**	**4241**	**0.37**	**0.37**	**0.31**	**0.42**	**0.29**	**0.53**	**0.28**	**0.34**	**0.38**	**0.31**	**0.47**
**3. User experience**	**5093**	**0.36**	**0.17**	**0.36**	**0.41**	**0.41**	**0.38**	**0.40**	**0.34**	**0.38**	**0.47**	**0.26**
**a. Client focus**	**5093**	**0.30**	**0.17**	**0.28**	**0.34**	**0.28**	**0.30**	**0.39**	**0.32**	**0.37**	**0.35**	**0.21**
System for discussing client preference	1279	0.83	0.82	0.78	0.84	0.82	0.82	0.78	0.71	0.89	0.86	0.95
Wait times less than one hour	4590	0.61	0.87	0.53	0.71	0.48	0.41	0.95	0.37	0.63	0.60	0.52
System for reviewing/reporting client opinion	4575	0.10	0.10	0.02	0.08	0.06	0.12	0.02	0.26	0.20	0.09	0.08
**b. Clear communication**	**5081**	**0.56**	**0.59**	**0.54**	**0.63**	**0.66**	**0.53**	**0.53**	**0.42**	**0.52**	**0.66**	**0.50**
ANC: Knowledge of delivery preparation	1902	0.91	0.86	0.93	0.90	0.99	0.75	0.88	0.90	0.99	1.00	0.95
ANC: Knowledge of side effects of iron	1404	0.43	0.56	0.54	0.45	0.74	0.25	0.19	0.08	0.66	0.59	0.21
ANC: Explanations on pregnancy danger signs/needed actions	2054	0.86	0.86	0.83	0.99	0.92	0.90	0.50	0.91	0.97	0.71	0.97
ANC: Knowledge of childbirth complications	2375	0.47	0.41	0.52	0.52	0.50	0.33	0.25	0.57	0.31	0.41	0.86
FP: Explanations on use of FP method	2183	0.69	0.69	0.65	0.70	0.74	0.70	0.42	0.73	0.58	0.89	0.82

FP: Explanations on FP side effects	2183	0.59	0.52	0.55	0.62	0.61	0.48	0.30	0.64	0.66	0.73	0.77
FP: Counseled on FP problems	2183	0.65	0.57	0.53	0.69	0.68	0.56	0.28	0.80	0.77	0.72	0.85
FP: Counseled on FP follow up visits	2183	0.93	0.94	0.90	0.94	0.93	0.97	0.85	0.92	0.93	0.93	0.95
SC: Counseled on diagnosis	4227	0.50	0.44	0.21	0.54	0.68	0.48	0.83	0.30	0.14	0.75	0.57
SC: Understand treatment and medications	3282	0.87	0.77	0.88	0.96	0.96		0.87		0.73	0.95	
SC: Food recommendations	4236	0.21	0.48	0.20	0.16	0.09	0.22	0.18	0.11	0.25	0.12	0.25

Data source: Service Provision Assessment Survey, 2007-2016. Data include primary care facilities, excluding hospitals. Scores were calculated by averaging the indicators for each quality subdomain and domain.

Abbreviations: ANC = Antenatal care, FP = Family planning, SC = Sick child, STI = Sexually-transmitted diseases, TB = Tuberculosis, HIV = Human immunodeficiency syndrome, NCD = Non-communicable diseases.

Appendix 7: Quality domains and subdomains by country

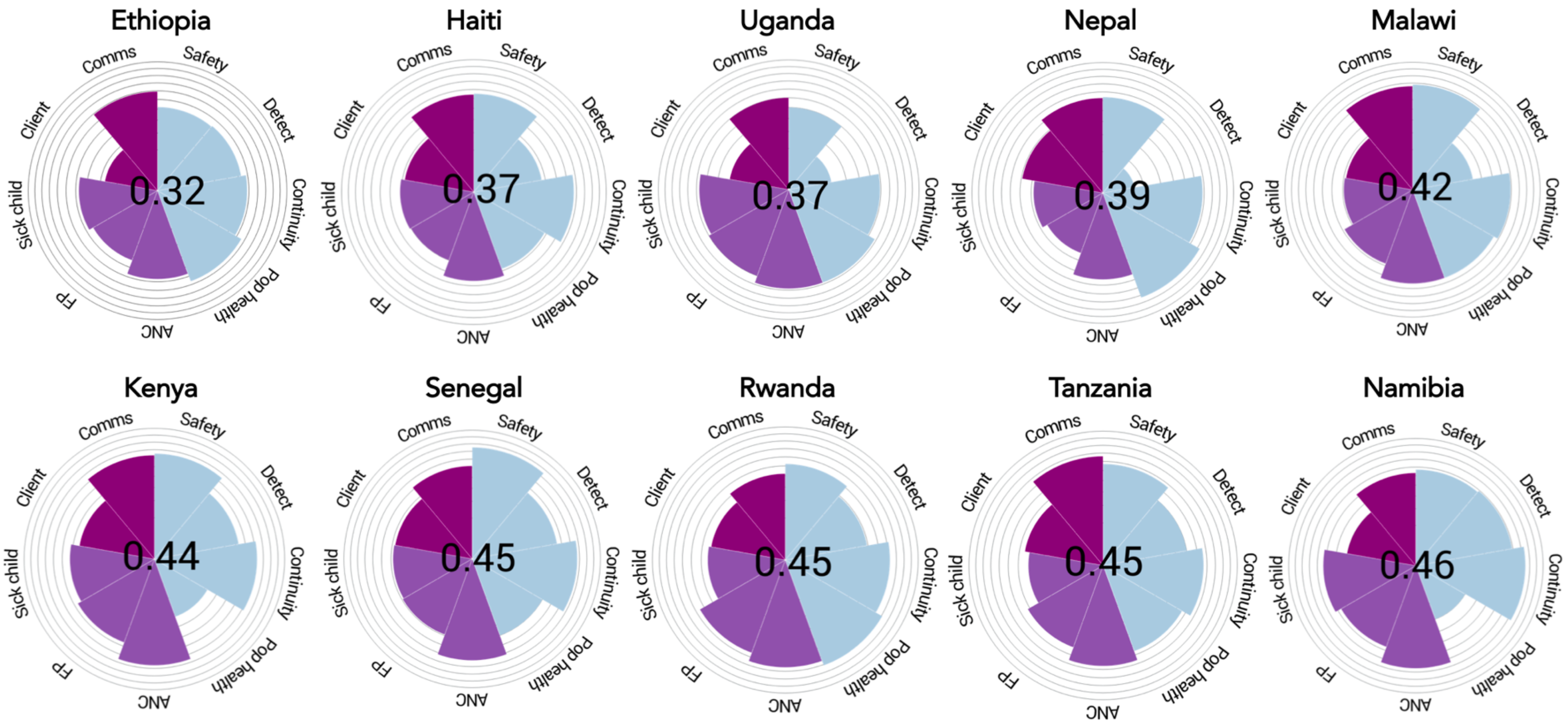

Each pie is one country sorted from lowest (left top) to highest (bottom right) quality scores
Numbers in each pie refer to the overall quality score for the country. Quality subdomains are shown as slices for each pie.
Quality domains are shown in colors – capable systems (blue), evidence-based care (purple), user experience (dark purple)
Each circular line is 0.1 quality score on a scale of 0 (lowest, center) to 1 (highest, periphery).
